# Prevalence of Oral Clefts among Live Births in Gansu Province, China

**DOI:** 10.3390/ijerph15020380

**Published:** 2018-02-23

**Authors:** Mengying Wang, Ruogu Meng, Zhuqing Wang, Dongjing Liu, Hui Huang, Chuyun Kang, Hongtian Li, Tao Wu, Siyan Zhan

**Affiliations:** 1 Department of Epidemiology and Biostatistics, School of Public Health, Peking University Health Science Center, Beijing 100191, China; mywang@bjmu.edu.cn (M.W.); ruogu_meng@bjmu.edu.cn (R.M.); wangzhuq@bjmu.edu.cn (Z.W.); yrldj@bjmu.edu.cn (D.L.); 1110306110@bjmu.edu.cn (H.H.); liht@bjmu.edu.cn (H.L.); siyan-zhan@bjmu.edu.cn (S.Z.); 2 Department of Child, Adolescent, and Women’s Health, School of Public Health, Peking University Health Science Center, Beijing 100191, China; kangchuyun@bjmu.edu.cn; 3 Institute of Reproductive and Child Health, Ministry of Health Key Laboratory of Reproductive Health, Peking University Health Science Center, Beijing 100191, China; 4 Office for National Maternal and Child Health Statistics of China, School of Public Health, Peking University Health Science Center, Beijing 100191, China

**Keywords:** oral clefts, live births, prevalence, cross-sectional study

## Abstract

*Background:* Oral clefts (OCs) are common human birth defects. Children with OCs in underdeveloped regions are more likely to suffer from poverty and hardship in their future lives. Here, we attempted to estimate the prevalence of OCs among live births in Gansu Province in 2008 to understand the epidemiologic pattern of the disease. *Methods:* A cross-sectional study was conducted from January 2008 to December 2008 in Gansu Province. The live births delivered between January and December 2008 with OCs were investigated through face-to-face questionnaire survey. *Results:* A total of 468 infants with OCs were identified among 347,137 live births in 2008 in Gansu Province, which yielded a prevalence of 1.35 per 1000 live births. The majority of these cases were CL (cleft lip) (prevalence = 0.85 per 1000 live births), and the prevalence of CLP (cleft lip and palate) and CP (cleft palate) was 0.34 and 0.11 per 1000 live births, respectively. We also found that the prevalence of OCs in Jiayuguan (3.39 per 1000 live births) and Dingxi (2.71 per 1000 live births) was higher than those of other cities in Gansu Province. Additionally, we failed to detect significant correlation between economic conditions of the cities and the prevalence of OCs in our study. *Conclusions:* The prevalence of OCs among live births in Gansu Province in 2008 was higher than the prevalence of OCs in other provinces in China. The high prevalence may reflect the need for further etiological studies to explore the potential risk factors in this region. In addition, more subtype information needs to be collected in future prevalence studies for better understanding of the epidemiologic pattern of the disease.

## 1. Introduction

Oral clefts (OCs) are among the most common congenital anomalies, typically including cleft lip (CL), cleft lip and palate (CLP), or cleft palate (CP) [1]. Children born with clefts may experience problems with speech, hearing, feeding, and psychosocial development, which brings a large burden to the patients and their families [2]. While most research efforts focus on exploration of genetic variations underlying the disease, understanding the OCs distribution is very important for estimating the disease burden and resource planning, as well as providing information for further etiological studies.

The prevalence of OCs varies with several factors, including gender, ethnicity, and environmental exposure and socioeconomic status [3,4,5]. Gansu Province is located in Northwest China, where the prevalence of OCs among live births has not been adequately studied. Since it is a medically underserved region, the prevalence of oral clefts in Gansu Province could be at a relatively high level. Even though several previous studies have reported the estimated prevalence of OCs among live births in Gansu Province [6,7], most of the analyses were performed using hospital-based surveillance data, which could be biased in such a developing area [8]. Therefore, a population-based survey of OCs may provide better information for disease distribution description and medical resources planning. Moreover, little is known about the distribution of the prevalence by different regions in Gansu Province.

In order to better understand the distribution of OCs in Gansu Province, we attempted to estimate the prevalence of OCs among live births in 2008 using the data collected in a population-based survey to illuminate the epidemiologic pattern of the disease.

## 2. Materials and Methods

### 2.1. Study Design and Participants

The present study was a retrospective analysis based on data collected in a two-stage population-based cross-sectional study, where all live births delivered between January 2008 and December 2008 were investigated by household questionnaire interview organized by the Department of Health in Gansu Province. The detailed information of the design and its conduct has been described previously [9]. In the first stage (January through July, 2008), oral cleft patients were identified and registered by trained local medical and public health workers at the township and village level in each city of Gansu Province based on the local residence registration and health records at the community level. At the second stage (August through December, 2008), based on the information collected in the first stage and the community level health records, the detailed information of the patients was investigated by trained local investigators. The organization, as well as the flow chart of the second stage investigation, is described in Figure S1, which presents the working scheme in rural and urban areas.

The inclusion criteria of participants were as follows: (1) OC patients born in 2008; (2) local registered residents or those living in Gansu Province for more than six months. Participants who did not consent to participate in the survey were excluded. All participants gave informed consent during the investigation.

### 2.2. Data Collection

Local investigators with medical knowledge were trained before the survey started. The investigators conducted the household questionnaire survey to collect information including demographic characteristics, family situation, affection status, and contact information of the patients. The information on clefts was recorded through visual inspection by clinicians in accordance with the International Classification of Diseases (ICD-9) diagnosis criteria, and classified the patients into three anatomical groups: CLP, CL, and CP [10]. However, syndromic and non-syndromic subtypes of oral clefts were not classified in the study.

### 2.3. Quality Control

Duplication and completeness of the questionnaires, as well as the reliability of the questionnaire information, were checked to ensure the quality of the data collected [9]. Duplicated cases were dropped after being identified by matching the name, sex, date of birth, and family address for records and checking the original questionnaire. Additionally, 5% of the records in each city were selected from the database using a systematic sampling method to evaluate the completeness of the questionnaires. To check the reliability of the information collected, three villages were selected where all the cases were interviewed for the second time. The concordance rate was 100%, and all the OCs cases were recorded in the original investigation.

In addition, the capture-recapture method was applied to estimate the completeness of case inclusion of this survey. The calculation formula is:

(1) N=[(M+1)×(n+1)/(m+1)]−1

(2) $$Capture\;rate=\frac{\left[M+\left(n-m\right)\right]}{N}\times 100\%$$

where N was the estimated number of cases; M and n were defined as the number of cases in our study and cases from another data source (the medical records in local hospitals in Gansu Province), respectively; m represented the common number of cases in both of the data sources. In the present study, the number of cases was 468 (M = 468), a total of 211 cases were identified from the medical records in local hospitals in Gansu Province (n = 211), all of which were included in our survey (m = 211), which yielded the capture rate of 100%.

### 2.4. Statistical Analysis

The prevalence of OCs among live births was defined as the number of patients with OCs who were born in 2008 divided by the total number of live births in the same year in different cities or in Gansu Province. The amount of total live births in Gansu Province was attained from the Annual Report of Gansu Province in 2009. The city-level aggregated data on live births of Gansu Province in 2008 were obtained from the Office for National Maternal and Child Health Statistics of China. In addition, we assessed the economic status of each city in Gansu Province based on per capita Gross Domestic Product (GDP), which was also attained from the Annual Report of Gansu Province in 2009. The 95% confidence interval for prevalence was calculated based on a Poisson distribution. Linear regression models were adopted to evaluate the correlation between economic conditions and the prevalence of OCs in cities. We performed all the data analysis using STATA (Version 13.0) (Stata-Corp LP., College Station, TX, USA).

## 3. Results

A total of 468 infants with OCs were identified among 347,137 live births in 2008 in Gansu Province, which yielded the prevalence of 1.35 per 1000 live births (95% CI: 1.23–1.48) (Table 1). Among 450 cases with subtype information (96.15% of the total cases), the majority subtype of these cases was CL (prevalence = 0.85 per 1000 live births). The prevalence of CLP and CP was 0.34 and 0.11 per 1000 live births, respectively (Table 1). As shown in Table 2, there were more male (61.75%) than female OC patients (38.25%). The majority of the OC patients were from rural areas (76.71%), among which 59.19% lived in national-level poverty counties.

**Figure 1:**
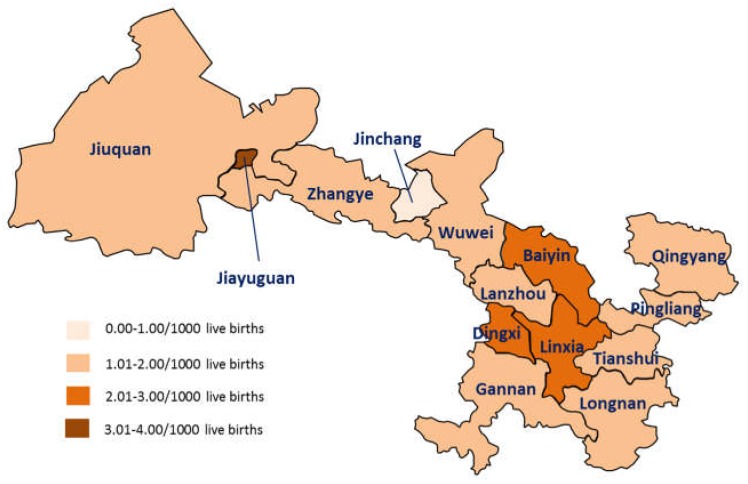
Prevalence of oral clefts in different cities in 2008 in Gansu Province.

The prevalence of OCs of different cities across Gansu Province is shown in Figure 1 and Table 3. The prevalence of OCs was the highest in Jiayuguan (3.39 per 1000 live births, 95% CI: 1.24–7.38), followed by Dingxi (2.71 per 1000 live births, 95% CI: 2.13–3.40), while the lowest prevalence was observed in Jinchang (0.42 per 1000 live births, 95% CI: 0.05–1.52). We failed to detect a correlation between GDP level and prevalence of OCs in cities (*p* = 0.34).

## 4. Discussion

The prevalence of OCs in the Gansu Province in Northwest China in 2008 was 1.35 per 1000 live births in the current population-based survey. We also found that the prevalence of OCs in Jiayuguan (3.39 per 1000 live births) and Dingxi (2.71 per 1000 live births) was higher than those of other cities in Gansu Province.

It has been reported that Asian populations, including Chinese, have a higher prevalence of OCs than other racial groups, such as Americans and Europeans [11]. Dai et al. found that the prevalence of OCs in Gansu Province had been increasing in recent years. In addition, a meta-analysis of the prevalence of OCs showed that there were no significant time trends of the disease in China [12,13]. Our study showed the prevalence of OCs in Gansu in 2008 was higher than those of other ethnic groups. The prevalence of OCs was 1.05 per 1000 live births among French and 0.85 per 1000 live births among Italians in 2008 [14], which was lower than the prevalence of OCs in our analysis.

The prevalence of OCs varied among different provinces and regions in China. Our study showed that prevalence of OCs among live births in Gansu Province in 2008 was higher than the reported prevalence of all OCs in other provinces in China, including Shaanxi Province in Northwest China (1.17 per 1000 live births) [15] and Beijing in North China (0.86 per 1000 live births) [16]. The high prevalence of OCs may reflect the high level of potential risk factors of the disease among the local populations, such as maternal smoking, alcohol consumption, and nutrition status during early pregnancy, which may be related to economic and social development conditions [1,17,18]. According to the National Bureau of Statistics of China in 2013, per capita Gross Domestic Product (GDP) of Gansu Province in 2008 (1789 dollars) was much lower than the national average (3,414 dollars) [19]. In addition, the national average number of Beds of Medical Institutions per 1000 Population was 3.05 in 2008 and 5.11 in 2015, both of which were higher than that of Gansu Province in 2008 (2.86 beds) and 2015 (4.91 beds) [20,21]. The current study may imply the situation of OCs in other medically underserved areas of similar social economic status with Gansu and benefit medical resources planning in China for better control and treatment of the disease.

The prevalence of OCs in our analysis was lower than the reported prevalence of OCs (1.46 per 1000 births) which was estimated using data from the birth defect monitoring network in Gansu Province in 2008 [12]. One potential reason may be that the birth defects monitoring data include stillbirth or abortion, while only live births were included in our study. It has been reported that stillbirths may have about three times higher prevalence of OCs than live births [22]. Data derived from the birth defect monitoring network were based on hospital monitoring samples, while the current study was a population-based survey investigating all OC cases, which could provide a better estimate of the prevalence avoiding referral bias usually influencing the reliability of hospital-based birth defect monitoring data [8].

The distribution of cleft types in this analysis was inconsistent with other previous studies. Dai et al. found that CLP was the most common cleft type (53.18%) followed by CL (31.39%) and CP (15.43%) using the hospital-based surveillance data [8]. However, the proportion of CL in our study (63.03%) was much higher than the reported proportion of total CL. In addition, 25.21% of the cases presented with CLP and 7.90% with CP in our study. CL is more visible than CP, and can be repaired within the first 6 months of life to assure normal sucking [23]. Therefore, the high prevalence of CL might have indicated that the access to special medical care for OCs in this region was quite limited, and that more financial support might improve the situation in Gansu Province.

The current study also found that the prevalence of OCs among live births varied across different cities in Gansu Province, where Jiayuguan and Dingxi were among the highest cities in terms of prevalence. Possible explanations might include the difference in the number of live births, as well as the potential risk factors of the disease, such as nutrition and smoking. Further studies are needed to further explore the etiology of OCs in the areas with high prevalence of the disease in Gansu Province. A study conducted by Dai et al. found a lower OC prevalence in urban areas compared with rural areas [8]. However, no significant correlation between economic conditions of the cities and the prevalence of OCs in our study was observed.

It has been reported that the ideal timing for cleft surgical repairs is between three and six months of age for lip repair, and between nine and 12 months of age for palate repair [24]. In addition, the surgical repair of OCs is a cost-effective intervention contributing not only to the improvement of patient function and quality of life, but also providing economic benefits to low- and middle-income countries [25,26,27]. However, many patients in low- and middle-income countries can only be repaired later in life—or not at all—due to lack of financial support [25,28]. In the current study, few cases of OCs were repaired. Therefore, further investigation of the surgical repair time among the patients in medically underserved areas such as Gansu Province would provide important information for medical resources planning.

The present study was a population-based cross-sectional study investigating the entire population in Gansu Province. In addition, the quality control procedure conducted through the investigation also ensured the data quality. The capture-recapture method was applied in evaluating the missing rate, which indicated that the study had identified all OC patients in this region in 2008. However, we did not describe the prevalence based on syndromic and non-syndromic subtypes, since no relevant data had been collected. Syndromic forms refer to those birth defects where the OCs are only part of some congenital syndromes while non-syndromic forms are isolated congenital malformation. Syndromic forms of OCs may require many more surgeries and more extensive interventions compared to the non-syndromic form. In future prevalence studies, a multi-disciplinary team, including orofacial clinical experts,will improve the data collection of important clinical and/or etiological information in such a population-based census of OCs.

## 5. Conclusions

We conducted a population-based survey to investigate all OC patients and describe the prevalence among live births in Gansu Province. The distribution of the prevalence varied across different regions of the province. More subtype information needs to be collected in further studies to better understand the epidemiological characteristics of this common congenital malformation in Northwest China.

## Figures and Tables

**Table 1:** The prevalence and relative proportions (%) of oral clefts in 2008 in Gansu province.

Subtype of Cleft	Number of Oral Clefts (%)	Prevalence ^a^ (95% CI)
Cleft lip only	295 (63.03)	0.85 (0.76–0.95)
Cleft lip and palate	118 (25.21)	0.34 (0.28–0.41)
Cleft palate only	37 (7.91)	0.11 (0.08–0.15)
Unknown ^b^	18 (3.85)	0.05 (0.03–0.08)
Total	468 (100.00)	1.35 (1.23–1.48)

^a^ Per 1000 live births; ^b^ The information about the subtype of clefts was missing or could not be identified.

**Table 2:** The distribution of oral clefts in 2008 in Gansu Province.

Characteristics	Number of Oral Clefts	Percentage (%)
Gender		
Male	289	61.75
Female	179	38.25
Area		
Town area	76	16.24
Rural area	359	76.71
Unknown ^a^	33	7.05
Economic status ^b^		
Poverty counties	277	59.19
Non-poverty counties	189	40.38
Unknown ^c^	2	0.43

^a^ The information about the residence of participants was missing or could not be identified; ^b^ According to directory of poverty-stricken counties in China; ^c^ The information about whether the participants was from poverty counties was missing or could not be identified.

**Table 3:** The prevalence of oral clefts in different cities in 2008 in Gansu Province.

Region	Number of Oral Clefts	Number of Live Births ^a^	Prevalence ^b^ (95% CI)
Jiayuguan	6	1769	3.39 (1.24–7.38)
Dingxi	74	27,318	2.71 (2.13–3.40)
Linxia	60	24,423	2.46 (1.87–3.16)
Baiyin	33	16,073	2.05 (1.41–2.88)
Lanzhou	55	28,854	1.91 (1.44–2.48)
Jiuquan	14	8094	1.73 (0.95–2.90)
Tianshui	61	35,897	1.70 (1.30–2.18)
Qingyang	44	26,800	1.64 (1.19–2.20)
Longnan	40	26,452	1.51 (1.08–2.06)
Pingliang	32	22,265	1.44 (0.98–2.03)
Gannan	13	9369	1.39 (0.74–2.37)
Wuwei	20	17,085	1.17 (0.72–1.81)
Zhangye	13	12,512	1.04 (0.55–1.78)
Jinchang	2	4758	0.42 (0.05–1.52)
Unknown ^c^	1	7809	0.13 (3.24 × 10^−3^–0.71)

^a^ Data source: number of live births in 2008 was from surveillance data collected by the Office for National Maternal and Child Health Statistics of China; ^b^ Per 1000 live births; ^c^ The information about which city the participants were from was missing or could not be identified.

## Supplementary Materials

The following are available online at http://www.mdpi.com/1660-4601/15/2/380/s1, Figure S1: Flow chart of the second stage investigation.
