# Supplementary material for: Prevalence of Oral Clefts among Live Births in Gansu Province, China
Source: Int J Environ Res Public Health. 2018 Feb 23;15(2):380. doi: 10.3390/ijerph15020380 (PMC5858449; doi:10.3390/ijerph15020380)
Supplement: Supplementary file 1 [file ijerph-15-00380-s001.docx]

Building leaders

PFPC leaders

Cities

Gansu Province

Sub-district office

PFPC of Districts

Group leaders

PFPC leaders

Villages and Towns Government

PFPC of Counties

PFPC of Cities

PFPC of Gansu Province

Health Department of Gansu Province

Districts

Counties

Sub-districts

Villages and Towns

Administrative villages

Communities

Groups (Natural villages)

Buildings

Houses

Houses

Self-management team leaders

Floor leaders

Supplementary Figure 1 Flow chart of the second stage investigation

Organizations responsible for the investigation;

Administrative division;

Investigators;

Lead investigators

Agreement signed between the organizations

**Figure S1**. please add the caption.
